# Joint Attention During Live Person-to-Person Contact Activates rTPJ, Including a Sub-Component Associated With Spontaneous Eye-to-Eye Contact

**DOI:** 10.3389/fnhum.2020.00201

**Published:** 2020-06-03

**Authors:** Swethasri Dravida, J. Adam Noah, Xian Zhang, Joy Hirsch

**Affiliations:** ^1^Interdepartmental Neuroscience Program, Yale School of Medicine, New Haven, CT, United States; ^2^Brain Function Laboratory, Department of Psychiatry, Yale School of Medicine, New Haven, CT, United States; ^3^Department of Neuroscience, Yale School of Medicine, New Haven, CT, United States; ^4^Department of Comparative Medicine, Yale School of Medicine, New Haven, CT, United States; ^5^Department of Medical Physics and Biomedical Engineering, University College London, London, United Kingdom

**Keywords:** joint attention, eye-to-eye contact, two-person neuroscience, live dyadic interactions, fNIRS, hyperscanning, eye tracking, neural coherence

## Abstract

Eye-to-eye contact is a spontaneous behavior between interacting partners that occurs naturally during social interactions. However, individuals differ with respect to eye gaze behaviors such as frequency of eye-to-eye contacts, and these variations may reflect underlying differences in social behavior in the population. While the use of eye signaling to indicate a shared object of attention in joint attention tasks has been well-studied, the effects of the natural variation in establishing eye contact during joint attention have not been isolated. Here, we investigate this question using a novel two-person joint attention task. Participants were not instructed regarding the use of eye contacts; thus all mutual eye contact events between interacting partners that occurred during the joint attention task were spontaneous and varied with respect to frequency. We predicted that joint attention systems would be modulated by differences in the social behavior across participant pairs, which could be measured by the frequency of eye contact behavior. We used functional near-infrared spectroscopy (fNIRS) hyperscanning and eye-tracking to measure the neural signals associated with joint attention in interacting dyads and to record the number of eye contact events between them. Participants engaged in a social joint attention task in which real partners used eye gaze to direct each other’s attention to specific targets. Findings were compared to a non-social joint attention task in which an LED cue directed both partners’ attention to the same target. The social joint attention condition showed greater activity in right temporoparietal junction than the non-social condition, replicating prior joint attention results. Eye-contact frequency modulated the joint attention activity, revealing bilateral activity in social and high level visual areas associated with partners who made more eye contact. Additionally, when the number of mutual eye contact events was used to classify each pair as either “high eye contact” or “low eye contact” dyads, cross-brain coherence analysis revealed greater coherence between high eye contact dyads than low eye contact dyads in these same areas. Together, findings suggest that variation in social behavior as measured by eye contact modulates activity in a subunit of the network associated with joint attention.

## Introduction

Eye contact is one of the most basic and prevalent behaviors that can occur between two individuals. Mutual eye contact can be used to indicate anything from attraction, attention, or aggression. The frequency of eye contacts can vary naturally based on the interaction; for example, partners who are comfortable with the topic they are discussing tend to make more eye contact than those who are uncomfortable (Argyle and Dean, 1965). Groups of individuals also make different amounts of eye contact; individuals with autism tend to make less eye contact than neurotypical individuals (Kanner, 1943, 1944) and tend to focus more on the mouth than the eyes when viewing faces (Klin et al., 2002; Neumann et al., 2006). While eye contact has been studied in social contexts (Hirsch et al., 2017; Koike et al., 2019b), few studies have focused on individual differences in eye contact behavior and the potential effect of these differences on known mechanisms of joint attention.

Joint Attention refers to the shared focus of two or more individuals on an object. A joint attention “event” refers to the alignment of attention due to a cue provided by an “initiator,” that is followed by one or more “responders.” Joint attention behaviors develop in early childhood, and the ability to engage in joint attention early in life is thought to be crucial to the development of language (Tomasello and Todd, 1983; Tomasello, 1988). Problems with initiating joint attention or responding to nonverbal joint attention initiated by another person (usually a parent) are some of the earliest observable deficits in children with autism (Dawson et al., 2004). Gaze-based joint attention between two people is a fundamental social communication, and the neural mechanisms of joint attention serve as an important clinical target in social disorders.

The majority of prior neuroimaging work in joint attention has focused on the neural mechanisms of single subjects in an fMRI scanner as they engage in joint attention tasks with an experimenter who is outside the scanner. These studies have identified a number of brain networks activated during joint attention tasks, including social cognitive networks as well as traditional attentional networks. In studies of social cognition, areas including the right temporoparietal junction (rTPJ) and medial prefrontal cortex (mPFC) show activity in response to mentalizing (Castelli et al., 2000; Frith and Frith, 2003) and other types of social perception and cognition (Hampton et al., 2008; Van Overwalle, 2009). Attentional tasks that do not involve a live partner show activity in parietal areas, and cardiovascular damage to these areas results in neglect, or the inability to direct the attention to one side of the visual field (Corbetta et al., 1995; Colby and Goldberg, 1999; Behrmann et al., 2004). A joint attention fMRI study using a virtual partner to direct and respond to the participant’s gaze showed increased activity in the mPFC when participants initiated joint attention with the virtual partner (Schilbach et al., 2010). Similar findings were observed in another fMRI study of participants engaging in joint attention with an experimenter where greater activity was observed in mPFC as well as right posterior superior temporal sulcus (pSTS), left pSTS, left inferior parietal lobe, right anterior insula, and right inferior frontal gyrus (Redcay et al., 2013). In these studies, data were acquired either from single individuals viewing a confederate or virtual avatar, or from two individuals who viewed videos of each other in real-time while lying in fMRI scanners. Here, we introduce both a live interaction paradigm to investigate joint attention and technology in which subjects can interact directly in a naturalistic environment with additional behavioral measurements of eye gaze.

Advancements in neuroimaging enable simultaneous acquisition of neural signals as well as behavioral measurements from interacting individuals who are seated face to face (Jiang et al., 2012; Pfeiffer et al., 2013; Babiloni and Astolfi, 2014; Hirsch et al., 2017, 2018; Piva et al., 2017). Functional near-infrared spectroscopy (fNIRS) is a neuroimaging tool that measures a blood oxygenation signal as a proxy for direct neuronal responses, similar to the BOLD signal of functional magnetic resonance imaging. However, the head-mounted optodes of fNIRS and the relative tolerance to small movements compared to fMRI enable simultaneous acquisition, hyperscanning, of neural signals from two face-to-face individuals as they interact. Hyperscanning using fNIRS permits investigation of theoretical questions under the framework of the Social Brain Hypothesis and the Dynamic Neural Coupling Hypothesis. The Social Brain hypothesis states that brains in interaction show different activity than brains performing the same task without live interaction (Di Paolo and De Jaegher, 2012; De Jaegher et al., 2016). In the context of joint attention, these hypotheses provoke the question: how do brains engaging in social joint attention with a partner differ from brains engaging in the same attentional task without live interaction? The Dynamic Neural Coupling hypothesis posits that brain areas across partners show covariation as they engage in tasks together that are due to the exchange of social information (Hasson and Frith, 2016). Thus, social joint attention is expected to engage unique cross-brain correlations compared to conditions in which both partners engage in non-social joint attention.

Many studies have looked at neural coherence between partners engaged in a task (Saito et al., 2010; Cui et al., 2012; Konvalinka and Roepstorff, 2012; Tanabe et al., 2012; Schilbach et al., 2013; Scholkmann et al., 2013; Hirsch et al., 2017, 2018). Hasson et al. (2012) have proposed that the mechanism of coherence between the brains of interacting individuals is driven by shared information; that is, some aspect of the interaction triggers similar activity in the two brains (Hasson and Frith, 2016). Measurement of neural coherence along with behavioral measures can serve as evidence that specific aspects of the interactions between partners leads to synchrony between their brain signals (Saito et al., 2010; Jiang et al., 2012, 2015). By recording the frequency of mutual eye contact events, we can relate “socialness” of joint attention, as measured by the numbers of mutual eye contact events, to coherence differences. Based on the neural coupling hypothesis that cross-brain neural synchrony reflects shared information between dyads, we hypothesize that these differences are driven, not just by the interaction, but by the number of eye contact events during the joint attention task.

Prior fMRI joint attention studies involve tasks in which subjects are explicitly asked to make direct gaze with a partner on a video screen prior to directing their attention. In this way, joint attention has typically been thought of as an extension of eye contact-based communication between the initiator of joint attention and the responder. In this conceptualization of a traditional joint attention event, the first step is direct eye gaze, followed by the initiator transferring his/her gaze to the attentional target. The final step occurs when the responder follows the initiator’s gaze to direct his/her attention to the target as well, resulting in joint attention. However, joint attention can also be achieved without initial direct eye gaze, as long as the responder follows the initiator’s gaze to focus on the target. Take the example of a passerby who notices someone else standing on the street and looking up at the sky. The first individual can follow the second’s gaze to look up at the sky as well, without having to first make eye contact. The extent to which individuals make eye contact without explicit instruction when engaging in joint attention may be related to their innate social behavior. Joint attention with and without un-instructed eye contact has not been investigated.

Here, we examine the role of social eye-to-eye contact behavior as a modulator of joint attention mechanisms to test the hypothesis that the two mechanisms are synergistic. Based on prior studies of eye contact, we have previously shown that direct, mutual eye contact engages the visual and social systems during social interaction (Hirsch et al., 2017; Noah et al., 2020). This predicts increased social exchange between participants who engage in increased eye contact, and these participants may recruit these areas more than in less social participants who make fewer eye contacts during a social joint attention task.

Pairs of participants were instructed to engage in joint attention tasks in which one person was assigned the role of initiator and the other person was the responder. The participants were not explicitly told to make eye contact, allowing for variation in the amount of eye contact across dyads. Importantly, performance on the joint attention task was at ceiling levels regardless of the number of mutual eye contact events between the partners, indicating that joint eye gaze was not required to correctly orient the attention. The effects of engaging in social joint attention were compared with non-social joint attention, in which both participants directed their attention to the same object without exchanging visual cues. Further, we examined how the number of eye contact events that occurred during the social runs modified the brain activity associated with social joint attention. In addition, cross-brain coherence between areas of the brain involved in face-processing was compared between high and low eye contact dyads. Our hypothesis was two-fold: first, that increased engagement in mutual eye contact would result in the modulation of social and high-level visual areas distinct from the neural responses to the joint attention task itself; and second, that pairs who engaged in more mutual eye contact would show greater cross-brain coherence between these areas.

## Materials and Methods

### Participants

Twenty-eight pairs of participants (mean age 29.4, 35 females) took part in the joint attention experiment. All participants completed the Edinborough Handedness Inventory (Oldfield, 1971), and fifty-four of the fifty-six participants were right-handed. There were twelve female-female, five male-male, and ten female-male pairs. One participant declined to report gender information. Participants had no known neurologic or psychiatric conditions. All participants provided written informed consent in accordance with the guidelines approved by the Yale University Human Investigation Committee (HIC # 1501015178) and were compensated for participating. Data were collected at the Yale School of Medicine, New Haven, Connecticut, United States. Prior to taking part in the joint attention experiment, each participant underwent a finger-thumb tapping procedure at a previous date.

### Paradigm

**FIGURE 1:**
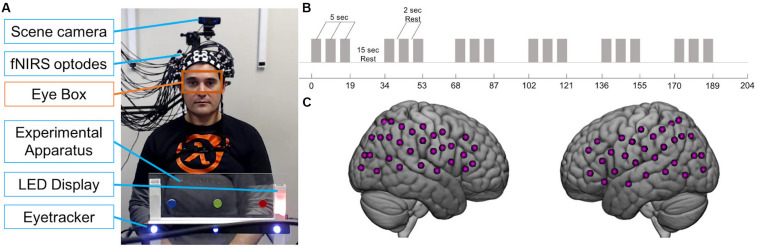
Joint attention experiment. **(A)** Example view of experimental apparatus during social trial in which partner seen in photo is the responder. Black bar with blue lights is the eye tracker. For each participant, an eye tracker was used to record eye movements and a scene camera was used to record what the participant saw. Orange box indicates eye box used to calculate mutual eye contact events. Photograph used with permission. **(B)** Paradigm time course. Gray bars represent 5-s trials. Task blocks consisted of three 5-s trials interspersed with 2-s of rest. 19-s task blocks alternated with 15-s rest blocks. **(C)** Example fNIRS 60-channel layout covering bilateral frontal, temporal, and parietal areas on one participant.

Participants were seated at a table across from each other, approximately 140 cm apart. Between them, on the table, was a transparent panel with two LEDs to the right side of each participant (Figure 1A). Each participant’s LEDs were not visible to their partner. The higher LED was either off or illuminated yellow, while the lower LED was either off or illuminated in red, green, or blue during the experiment. In the center of the panel were three circular stickers, equally spaced, one red, one green, and one blue. Importantly, the position of each color was the same in space for each participant; for example, if the red sticker was to the right for Subject 1, it was to the left for Subject 2 sitting across from Subject 1, so that when both subjects looked at the red sticker, they were looking at the same point in space. Below the stickers was a white bar with a magnet on which the Tobii x3-120 eye tracker was mounted (see Figure 1A). The panel did not obstruct the partners’ view of each other’s faces. A scene camera was positioned on a camera mount attached to an articulated arm behind each participant and aimed to record what the participant was seeing during the experiment. LEDs were controlled by serial port commands sent from a Python script to an Arduino board that was pre-programmed to recognize commands as “on” and “off” for each color.

### Task

**FIGURE 2:**
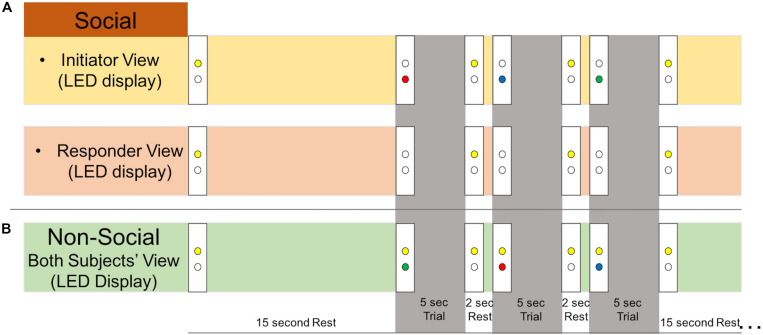
Experimental stimuli. Example LED display as viewed by each subject during social and non-social runs. During all rest periods, participants were instructed to look at the lit yellow LED. An auditory tone indicated to both participants that the trial was over and to look back at the yellow LED rest cue. **(A)** During 5-s social trials, the initiator would receive an LED cue (green, blue, or red) and use his/her eye gaze to indicate the location of the correct target to the responder. The responder would not receive an LED cue during the trial. **(B)** During 5-s non-social trials, both participants received an LED cue (green, blue, or red) indicating the correct gaze target. The yellow LED remained on during the whole run to remind participants that it was non-social.

There were two types of runs: “social runs” in which one subject initiated the joint attention events and the other subject responded, and “non-social runs” in which both subjects received an LED cue that directed their attention. Subjects were told that as a dyad, the goal of the task was for both participants to gaze at the correct target. For the social runs, the initiator was told that his/her goal was to show his/her partner the correct target using only eye movements. The responder was told to use his/her partner’s gaze to find the correct target. For the non-social runs, subjects were told to look at the correct target, shown by the LED color. Runs began with both yellow LEDs illuminated for the first 5 s, during which each participant focused their gaze on their yellow LED. Each run consisted of six 19-s task blocks and six 15-s rest blocks (Figure 1B). Task blocks included three 5-s attention trials interspersed with 2-s of rest. Example LED displays during the rest and task blocks are shown in Figure 2. During the joint attention trials in the social runs, a colored LED to the right of the apparatus indicated the target to the initiator. The initiator was instructed to communicate the location of the target to the responder non-verbally, using only eye movements. Importantly, the initiator was not told to first make eye contact with the responder before directing his/her gaze to the target. An auditory beep indicated the trial was over and both participants should return their gaze to the yellow LED, which was illuminated during rest periods. When the next trial started, the yellow LEDs would become unlit and the initiator would see the colored LED with the next target, while the responder would not see any LED cue. During all rest periods, participants were instructed to focus their gaze on the lit, yellow LED to the right of the apparatus. The yellow LED was also illuminated during the entirety of the non-social joint attention runs. During these runs, both participants received the same colored LED light cueing them to the correct target.

There were four social runs total: two runs in which Subject A was the initiator and two runs in which Subject B was the initiator. There were also two non-social joint attention runs with no initiator or responder; therefore, there were six runs total. The order of the runs was randomized and each type was completed once before any were repeated. For example, one possible run order was: Subject A initiates, Subject B initiates, non-social, Subject A initiates, Subject B initiates, non-social. Prior to the start of the recording, participants engaged in a “practice run,” in which they experienced two blocks of each type (Subject A initiating, Subject B initiating, non-social). The experimenter observed and talked to the participants during the practice run, ensuring that both participants understood the task and could see the targets, the LED and each other, and reminding them to keep their heads as still as possible. Once the experiment began, participants wore the instruments the entire time but had about a 15-s break between runs, when the fNIRS and eye tracking recordings were stopped and re-started.

### Eye Tracking

Eye tracking data were acquired using two Tobii Pro x3-120 eye trackers (Tobii Pro, Stockholm, Sweden), one per participant, at a sampling rate of 120 Hz. Eye trackers were mounted on the experimental apparatus facing each subject (Figure 1A). Each subject was calibrated using a three-point calibration on their partner’s face prior to the start of the experiment. The partner was instructed to stay still and look straight ahead while the subject was told to look first at the partner’s right eye, then left eye, then the tip of the chin.

UDP signals were used to synchronize the triggers from the stimulus presentation program to a custom virtual keyboard interpretation tool written in Python to the Tobii Pro Lab software. When a joint attention trial started and ended, UDP triggers were sent wirelessly from the paradigm computer to the eye tracking computers, and the virtual keyboard “typed” a letter that marked the events in the eye tracking data recorded in Tobii Pro Lab.

### Functional Near-Infrared Spectroscopy Acquisition

Functional NIRS signals were acquired from two participants simultaneously using a continuous wave Shimadzu LABNIRS system (Shimadzu Corp., Kyoto, Japan) with eighty optodes. Three wavelengths of light (780, 805, and 830 nm) were emitted by each emitter and the temporal resolution of acquisition was 27 ms. Light absorption measured by each detector was converted to concentrations of oxyhemoglobin (oxyHb) and deoxyhemoglobin (deoxyHb) using a modified Beer-Lambert equation. Twenty emitters and twenty detectors were placed on each participant to cover bilateral frontal, temporal, and parietal areas (Table 1) resulting in 60 channels per head (Figure 1C). Participants were fitted with custom caps with optode separation distances of either 2.75 or 3 cm for participants with head circumferences less than 56.5 cm or greater than 56.5 cm, respectively. Caps were placed such that the front-most optodes were approximately 1 cm above nasion, to maximize similar positioning across participants. A lighted fiberoptic probe (Daiso, Hiroshima, Japan) was used to remove hair from each optode holder before placing the optode to ensure contact with the skin. A TTL triggering system was used to synchronize triggers sent from the Python stimulus presentation script to the fNIRS system.

**TABLE 1:** Median channel MNI coordinates for each of the 60 channels with anatomical labels and probabilities.

Channel	*X*	*Y*	*Z*	BA	Anatomical Area	Probability
1	−43.67	−57.33	59.33	7	Somatosensory Association Cortex	0.305
				40	Supramarginal gyrus part of Wernicke’s area	0.683
2	−51.67	−52.00	56.00	40	Supramarginal gyrus part of Wernicke’s area	1.000
3	−47.33	−74.67	42.33	19	V3	0.237
				39	Angular gyrus, part of Wernicke’s area	0.644
4	−49.67	26.67	38.67	9	Dorsolateral prefrontal cortex	0.677
5	−59.00	2.00	40.67	6	Pre-Motor and Supplementary Motor Cortex	0.832
6	−61.33	−20.67	47.67	1	Primary Somatosensory Cortex	0.257
				2	Primary Somatosensory Cortex	0.268
				6	Pre-Motor and Supplementary Motor Cortex	0.217
7	−61.33	−40.67	49.67	40	Supramarginal gyrus part of Wernicke’s area	0.989
8	−56.33	−65.33	39.33	39	Angular gyrus, part of Wernicke’s area	0.625
				40	Supramarginal gyrus part of Wernicke’s area	0.375
9	−49.00	37.67	27.33	46	Dorsolateral prefrontal cortex	0.947
10	−60.33	13.33	25.33	9	Dorsolateral prefrontal cortex	0.476
				44	pars opercularis, part of Broca’s area	0.231
				45	pars triangularis Broca’s area	0.231
11	−64.67	−9.67	36.67	6	Pre-Motor and Supplementary Motor Cortex	0.720
12	−66.00	−30.67	41.67	40	Supramarginal gyrus part of Wernicke’s area	0.627
13	−62.00	−55.33	35.67	40	Supramarginal gyrus part of Wernicke’s area	0.837
14	−49.67	−81.33	23.33	19	V3	0.464
				39	Angular gyrus, part of Wernicke’s area	0.536
15	−58.33	25.67	14.67	45	pars triangularis Broca’s area	0.709
16	−65.00	0.67	20.33	6	Pre-Motor and Supplementary Motor Cortex	0.592
17	−68.00	−19.33	27.67	2	Primary Somatosensory Cortex	0.234
				40	Supramarginal gyrus part of Wernicke’s area	0.228
18	−67.00	−43.33	29.67	40	Supramarginal gyrus part of Wernicke’s area	0.962
19	−54.00	−76.00	19.00	19	V3	0.495
				39	Angular gyrus, part of Wernicke’s area	0.505
20	−55.33	35.67	5.67	45	pars triangularis Broca’s area	0.393
				46	Dorsolateral prefrontal cortex	0.290
				47	Inferior prefrontal gyrus	0.281
21	−61.67	5.33	1.67	22	Superior Temporal Gyrus	0.638
22	−68.00	−11.33	13.33	22	Superior Temporal Gyrus	0.259
				42	Primary and Auditory Association Cortex	0.316
				43	Subcentral area	0.378
23	−69.33	−34.33	16.67	22	Superior Temporal Gyrus	0.558
				42	Primary and Auditory Association Cortex	0.242
24	−66.00	−56.33	11.67	21	Middle Temporal gyrus	0.381
				22	Superior Temporal Gyrus	0.576
25	−48.00	−86.67	7.00	19	V3	0.873
26	−53.67	17.33	−9.33	38	Temporopolar area	0.566
				47	Inferior prefrontal gyrus	0.315
27	−67.67	−6.33	−10.67	21	Middle Temporal gyrus	1.000
28	−71.00	−24.67	−1.67	21	Middle Temporal gyrus	0.627
				22	Superior Temporal Gyrus	0.264
29	−69.67	−46.00	2.00	21	Middle Temporal gyrus	0.585
				22	Superior Temporal Gyrus	0.405
30	−60.00	−69.00	0.00	19	V3	0.306
				37	Occipito-temporal cortex	0.516
31	46.67	−62.33	55.67	7	Somatosensory Association Cortex	0.491
				40	Supramarginal gyrus part of Wernicke’s area	0.509
32	48.00	−74.67	40.00	19	V3	0.351
				39	Angular gyrus, part of Wernicke’s area	0.570
33	54.67	−53.33	54.33	40	Supramarginal gyrus part of Wernicke’s area	1.000
34	56.67	−65.33	37.67	39	Angular gyrus, part of Wernicke’s area	0.673
				40	Supramarginal gyrus part of Wernicke’s area	0.327
35	61.67	−42.33	50.67	40	Supramarginal gyrus part of Wernicke’s area	1.000
36	63.67	−19.67	48.33	1	Primary Somatosensory Cortex	0.259
				2	Primary Somatosensory Cortex	0.204
				3	Primary Somatosensory Cortex	0.208
				6	Pre-Motor and Supplementary Motor Cortex	0.223
37	61.00	3.67	41.67	6	Pre-Motor and Supplementary Motor Cortex	0.750
38	52.00	28.67	37.67	9	Dorsolateral prefrontal cortex	0.672
				46	Dorsolateral prefrontal cortex	0.229
39	46.67	−84.67	19.67	19	V3	0.852
40	62.67	−57.67	33.33	39	Angular gyrus, part of Wernicke’s area	0.287
				40	Supramarginal gyrus part of Wernicke’s area	0.713
41	68.00	−32.00	42.00	40	Supramarginal gyrus part of Wernicke’s area	0.694
42	67.33	−6.67	34.67	6	Pre-Motor and Supplementary Motor Cortex	0.854
43	62.67	14.33	25.67	9	Dorsolateral prefrontal cortex	0.528
				45	pars triangularis Broca’s area	0.260
44	51.33	38.33	28.33	46	Dorsolateral prefrontal cortex	0.867
45	54.00	−75.33	19.33	19	V3	0.386
				39	Angular gyrus, part of Wernicke’s area	0.614
46	68.67	−44.00	27.00	40	Supramarginal gyrus part of Wernicke’s area	0.821
47	70.00	−20.67	28.33	2	Primary Somatosensory Cortex	0.209
				40	Supramarginal gyrus part of Wernicke’s area	0.348
48	67.67	2.67	21.33	6	Pre-Motor and Supplementary Motor Cortex	0.618
49	60.67	26.67	17.33	45	pars triangularis Broca’s area	0.638
				46	Dorsolateral prefrontal cortex	0.299
50	48.00	−86.33	3.33	18	Visual Association Cortex (V2)	0.254
				19	V3	0.746
51	66.00	−57.33	10.67	21	Middle Temporal gyrus	0.387
				22	Superior Temporal Gyrus	0.470
52	72.00	−33.67	15.33	22	Superior Temporal Gyrus	0.541
				42	Primary and Auditory Association Cortex	0.325
53	70.00	−10.67	12.33	22	Superior Temporal Gyrus	0.319
				42	Primary and Auditory Association Cortex	0.300
				43	Subcentral area	0.322
54	62.67	11.67	5.67	22	Superior Temporal Gyrus	0.364
				44	pars opercularis, part of Broca’s area	0.358
55	58.00	35.33	9.67	45	pars triangularis Broca’s area	0.440
				46	Dorsolateral prefrontal cortex	0.467
56	59.00	−69.67	−2.67	19	V3	0.460
				37	Occipito-temporal cortex	0.507
57	71.00	−46.33	0.33	21	Middle Temporal gyrus	0.623
				22	Superior Temporal Gyrus	0.335
58	73.00	−23.67	−0.33	21	Middle Temporal gyrus	0.458
				22	Superior Temporal Gyrus	0.399
59	69.00	−4.67	−7.33	21	Middle Temporal gyrus	0.921
60	57.67	22.67	−1.33	45	pars triangularis Broca’s area	0.237
				47	Inferior prefrontal gyrus	0.595

*Negative x-coordinate indicates the left side. MNI coordinates were converted to Tailarach coordinates to generate cluster labels. Note that for the first ten pairs of participants, optodes were arranged in a 58 channel layout. The layout was switched to cover more of the higher-level visual cortex.*

### Digitization

A Polhemus Patriot digitizer (Polhemus, Colchester, VT, United States) was used to record the position of each optode relative to each subject’s anatomical landmarks (nasion, inion, Cz, left pre-auricular point, right pre-auricular point). Coordinates were converted into Montreal Neurological Institute (MNI) space using NIRS-SPM (Ye et al., 2009). MNI coordinates and anatomical labels for the median channel locations across all subjects are listed in Table 1.

### Analysis of Eye Gaze Position

Tobii Pro Lab software (Tobii Pro, Stockholm, Sweden) was used to create areas of interest for subsequent eye tracking analyses run in MATLAB 2014a (Mathworks, Natick, MA, United States). The first area of interest was the “eye box” (see Figure 1A), which covered the portion of the face of the partner in the video to which the participant’s eye tracking was targeted during initiation and response to joint attention events. The second area of interest was the “target box” which covered the part of the scene where the participant’s gaze rested on the red, blue, and green targets. Areas of interest were created manually based on watching the eye tracking videos. Data were exported from Tobii and custom scripts in MATLAB were used calculate the behavioral measures of mutual eye contact events, accuracy, and latency to targets. An “eye hit” was defined when the participant’s gaze was directed to the eye box of his/her partner. A mutual eye contact event was defined as simultaneous eye hits for both partners lasting at least 10 frames (83 ms). The videos of each participant recorded using the eye tracking software were also viewed to confirm that the participants’ head movement was minimal during the task.

The median number of eye contact events for all participant pairs was used to categorize dyads into two groups: “high eye contact pairs” and “low eye contacts pairs” whose number of mutual eye contact events were above and below the median, respectively. The interaction between groups (high and low eye contact pairs) and behavioral measures (latency to target and duration on target) was examined using a two-way repeated measures ANOVA. *Post hoc* tests applied Tukey’s multiple comparisons test with Bonferroni correction. The amount of cross-brain coherence between dyads in the high and low eye contact groups was compared using wavelet coherence analysis (see below).

### Functional Near-Infrared Spectroscopy Analysis

Analysis methods for fNIRS data were similar to those previously reported and are explained in detail elsewhere (Dravida et al., 2017; Hirsch et al., 2017, 2018; Noah et al., 2017, 2020). The signal used for the analysis was the combined hemoglobin signal, the sum of the oxyhemoglobin and the inverted deoxyhemoglobin signals. Briefly, wavelet detrending was used to remove baseline drift. Noisy channels were removed automatically if the root mean square of the signal was more than 10 times the average for that participant. A principal component analysis (PCA) spatial filter was used to remove physiologic, non-neural components from the signal (Zhang et al., 2016, 2017). For each run, a general linear model (GLM) of the joint attention task paradigm (Figure 1B) convolved with a canonical hemodynamic response function was used to generate beta values for each channel. Data from each participant were then registered to the median channel locations for all participants and reshaped into 3D voxel-based images with 3,753 standard 2x2x2 mm^3^ voxels. Thus, the beta values in each channel for each participant represent the fit of the individual participant’s data to the model. The first-level model resulted in contrast images for the Social > Non-social joint attention contrast, Initiation of joint attention > Non-social joint attention, and Response to joint attention > Non-social joint attention for each participant.

SPM8 (Wellcome Trust, London, United Kingdom) was used for second-level, group contrast analysis and *t*-tests were used to generate statistical maps of brain activity, using a threshold of *p* < 0.01. For each of the contrasts above, in the second-level group analysis, the average number of eye contacts in all four social runs for each participant was added as a covariate. Thus the Social>Non-Social main effect contrast is the result of the canonical regressor with all ones in the design matrix. The contrast with the covariate regressor answers the question of how the activity due to social joint attention changes more in participants who made more eye contact than in participants who made less eye contact (modulatory effect of eye contact on joint attention).

An alternate analysis was run using only the main effect regressor. Separating the participants into “high eye contact dyads” and “low eye contact dyads,” the contrast of High eye contact (Social > Non-social) > Low eye contact (Social > Non-social) was computed to further illustrate the effect of the high eye contact dyads’ behavior on the activity due to the joint attention task.

### Wavelet Coherence Analysis

Coherence analyses were performed on the deoxyhemoglobin signals rather than the oxyhemoglobin or combined signals due to the increased sensitivity of the oxyhemoglobin to systemic signal components (Franceschini et al., 2004; Huppert et al., 2006; Kirilina et al., 2012; Tachtsidis and Scholkmann, 2016; Zhang et al., 2016) which affect residual signals used in the analysis. Details on this method can be found in Hirsch et al. (2017, 2018), Piva et al. (2017). Briefly, channels were grouped into 12 anatomical regions and wavelet coherence analysis was evaluated between all groups across participants in a pair exhaustively. The wavelet coherence analysis decomposes time-varying signals into their frequency components. Here, the wavelet kernel used was a complex Gaussian (“Cgau2”) provided in MATLAB. The residual signal from the entire data trace was used, with the activity due to the task removed, similar to traditional PPI analysis (Friston et al., 1997). Sixteen scales were used and the range of frequencies was 0.1 to 0.025 Hz. Based on prior work, we restricted the wavelengths used to only those that reflect fluctuations in the range of the hemodynamic response function; coherence results in the range higher than 0.1 Hz have been shown to be due to non-neural physiologic components (Nozawa et al., 2016; Zhang et al., 2020). Therefore, 11 wavelengths total were used for the analysis. Complex coherence values were averaged because averaging complex coherence values results in greater correlation between measured and expected coherence compared to averaging the absolute value of coherence over time (Zhang et al., 2020).

Cross-brain coherence is the correlation between the corresponding frequency components across interacting partners, averaged across all time points and represented as a function of the wavelength of the frequency components. The difference in coherence between dyads in the high eye contact group and dyads in the low eye contact group was measured using *t*-tests for each frequency component. Only wavelengths shorter than 30 s were considered. All four social runs were used to find pairs of areas across participants in a dyad that showed significant coherence difference between the high and low eye contact groups. An analysis on shuffled pairs of participants who did not do the task together was conducted in order to report coherence that was specific to the pair interaction and not due to engagement in a similar task. Results that showed increased coherence in the group of high eye contact dyads relative to the group of low eye contact dyads for at least three consecutive wavelengths are reported.

## Results

### Behavioral Eye Tracking Results

**FIGURE 3:**
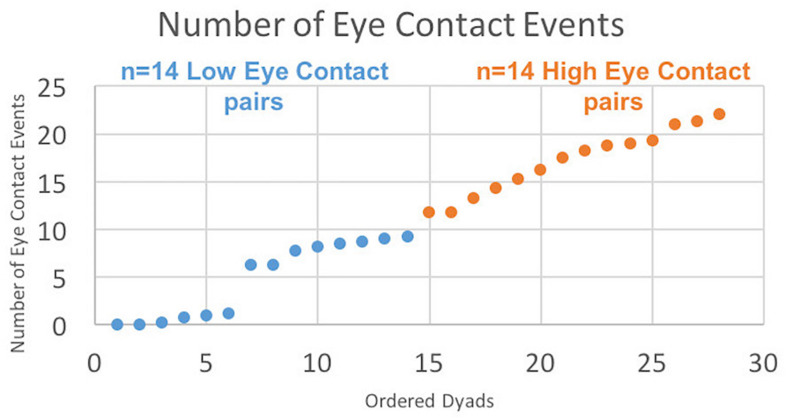
Number of eye contact events for each pair ordered. “Eye contact event” is defined when both subjects’ eye gaze is located in the partner’s eye box (see Figure 1B) for at least 10 frames (83 ms). Twenty-eight dyads are ordered on the *x*-axis from least to most number of eye contact events, with median number of eye contact events = 10.5. Blue points constitute the “Low Eye Contact Dyads;” orange points constitute the “High Eye Contact Dyads.” For the within-brain, general linear model analysis, the number of eye contact events for each participant was included as a second-level covariate in the group analysis, regardless of participant group. For the cross-brain coherence, the high and low eye contact groupings were used.

**FIGURE 4:**
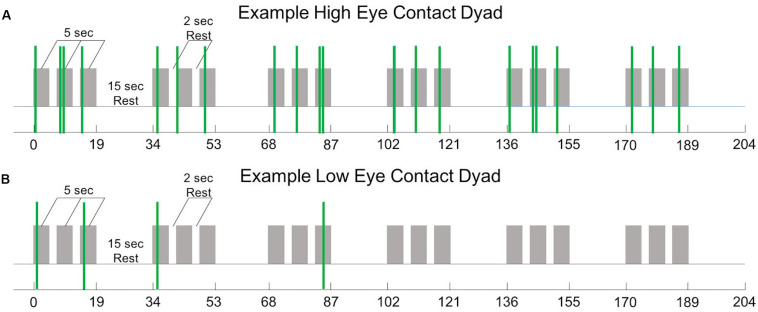
**(A)** Experiment time course (see Figure 1A) with 22 mutual eye contact events (green bars) from one social run from one “high eye contact” dyad. **(B)** Experiment time course with 4 mutual eye contact events (green bars) from one social run from one “low eye contact” dyad.

All mutual eye contact events that occurred from the start to the end of the run were counted. Most eye contact events occurred before joint attention to the target; however, for some dyads, some eye contact events occurred during the trial but after joint gaze on the target was achieved, resulting in greater than 18 events. No eye contact events occurred during rest periods. Dyads were ordered (Figure 3, *x*-axis) from least to most number of eye contact events (Figure 3, *y*-axis). The median number of mutual eye contact events across all dyads was 10.5. Pairs with greater than 10.5 eye contact events were classified as “high eye contact dyads” (orange circles) and pairs with lower than 10.5 eye contact events were classified as “low eye contact dyads” (blue circles). Onset of eye contact events during one social run for an example high eye contact dyad (Figure 4A) and a low eye contact dyad (Figure 4B) are shown. During social runs, the high eye contact dyads had an average of 17.1 eye contacts events, while the low eye contact dyads had an average of 4.8 eye contact events. There were no mutual eye contact events during the non-social runs for any of the dyads. High eye contact dyads consisted of 7 female-female pairs, 2 male-male pairs, and 5 male-female pairs. Low eye contact dyads consisted of 5 female-female pairs, 3 male-male pairs, 5 male-female pairs, and one pair in which a participant declined to report gender information. Thus the differences that are reported between the high and low eye contact dyads cannot be explained by the demographic compositions of the groups.

Eye tracking data were used to quantify the latency to targets, accuracy, and number of eye hits on the partner’s face for each participant. The average latency to target was 952.6 ms. Average latency to target was lowest during the non-social attention trials, when the subject’s attention was directed using an LED. There was no difference in latency to target between the three colored targets regardless of condition. Accuracy to target was 100% in both groups regardless of condition; only one subject made an error on one non-social trial by failing to redirect their attention on the next trial at the auditory cue.

The effect of the interaction between groups (high and low eye contact pairs) and conditions (initiation, response, and non-social attention) on the latency to target was determined using a two-way repeated measures ANOVA, with *post hoc* tests using Tukey’s multiple comparisons test with Bonferroni correction. As expected, there was a significant effect of condition (*p* < 0.0001); i.e., latency to target was significantly slower when the participant was the responder than during he/she was the initiator or when he/she was in a non-social run (Supplementary Figure S1). There was also a significant effect of group, with higher latencies in the high eye contact group than the low eye contact group (*p* = 0.0092). There was no significant group by condition interaction.

Each video was watched by an examiner who recorded the location of the eye box and whether or not the location of the eyes moved from the start to the end of the video. Minimal head movement was observed during the task in all eye tracking videos, regardless of whether the participant was part of the low or high eye contact pairs.

### Within-Subject General Linear Model Results

**FIGURE 5:**
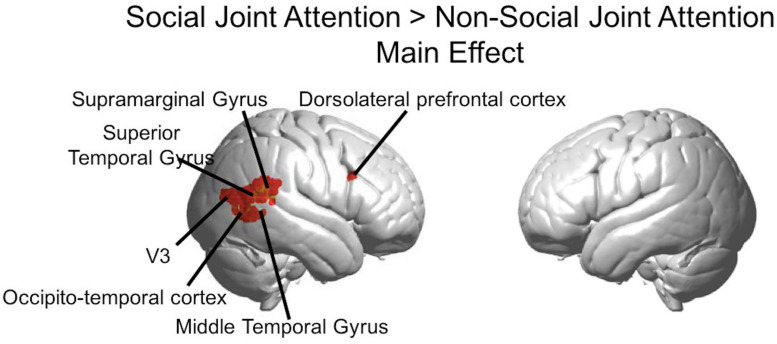
Group GLM Results: Social joint attention effect. Whole brain rendered images showing greater activity for Social > Non-social joint attention (*p* < 0.01, uncorrected). Social conditions include all runs in which one subject was an initiator and one was a responder. Results are combined across all participants (*n* = 56).

**FIGURE 6:**
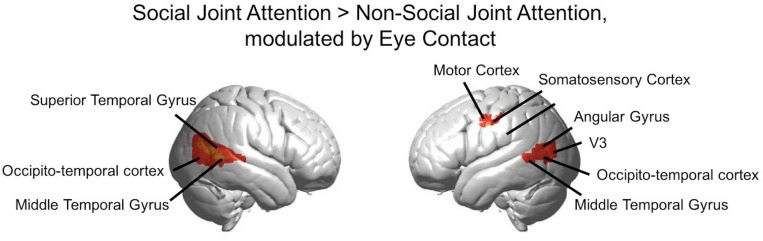
Group GLM Results: Social joint attention modulated by eye contact. Red-yellow areas on whole brain rendered images indicate greater activity in the Social > Non-social joint attention with greater activity in participants who made more eye contact (*p* < 0.01, uncorrected; *n* = 56).

Results of the joint attention contrasts are presented with and without the effect of the mutual eye contact events between partners. The GLM main effect figure (Figure 5) represents the results of the main task effect for the Social joint attention> Non-social joint attention contrast. This is the result of the main effect of the canonical regressor from the column of ones in the design matrix. The results are also presented using the covariate regressor of the number of eye contact events in the second-level analysis for the Social > Non-social joint attention contrast (Figure 6). In other words, Figure 5 displays the result of the social joint attention task for all pairs, while Figure 6 represent the results of the modulatory effect of eye contact on joint attention.

The Social > Non-social main effect (orthogonal to the modulatory effect of mutual eye contact) revealed activity in the following clusters (*p* < 0.01; Figure 5): right superior temporal gyrus/supramarginal gyrus (n of voxels = 160, peak T = 3.87, peak MNI coordinate of (70, −42, 14)), right occipito-temporal cortex (n of voxels = 18, peak T = 3.05, peak MNI coordinate at (58, −70,6)), and right dorsolateral prefrontal cortex (n of voxels = 14, peak T = 2.89, peak MNI coordinate of (58, 18, 28)). These findings are in line with expected result for joint attention tasks.

**FIGURE 7:**
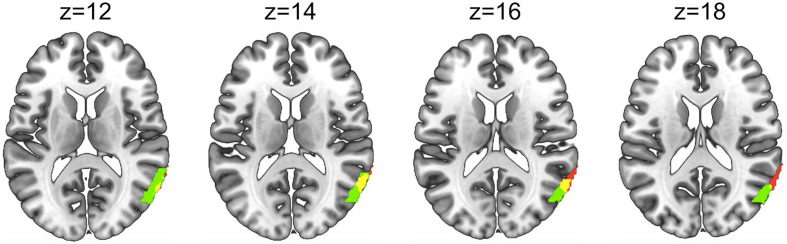
Overlap (yellow) between the area showing greater activity for the Social>Non-social joint attention main effect in Figure 5 (red) and the Social > Non-social joint attention modulated by eye contact in Figure 6 (green). Areas and overlap are displayed on four axial slices using a template brain.

The rendered hemodynamic results of the Social > Non-social contrast with the group-level eye contact covariate regressor are shown in Figure 6, with red-yellow clusters denoting higher activity with more eye contact events (*p* < 0.01). This resulted in a cluster on the left consisting of angular gyrus, middle temporal gyrus, occipito-temporal cortex, and V3 (n of voxels = 147, peak t = 3.76, peak MNI coordinate of (−58, −70, 12)) and a cluster on the right comprised of the superior temporal gyrus, middle temporal gyrus, and occipito-temporal cortex (n of voxels = 671, peak t = 3.81, peak MNI coordinate of (62, −50, 6)), as well as smaller clusters in the left motor cortex (see Table 2 for details). There are 61 voxels in the right superior temporal gyrus/middle temporal gyrus/occipito-temporal cortex area that overlap between the results shown in Figures 5, 6. The area of overlap is shown in Figure 7 and the brain regions covered by this area are listed in Table 3. The contrast of the social joint attention task relative to rest resulted in similar areas as above, with and without the modulation of eye contact, respectively (Supplementary Figure S2).

**TABLE 2:** Group-level general linear model contrast results (*p* < 0.01).

Peak MNI coordinates	Peak T	*P*	# Voxels	BA	Anatomical Area	Probability
**Social** > **Non-social Joint Attention (Task effect)**						
(70, −42, 14)	3.87	0.00015	160	22	Superior Temporal Gyrus	0.636
				40	Supramarginal Gyrus	0.171
(64, −60, 4)	3.38	0.00068	31	37	Occipito-temporal cortex	0.337
				21	Middle Temporal Gyrus	0.300
				22	Superior Temporal Gyrus	0.180
(58, −70, 6)	3.05	0.00177	18	19	V3	0.426
				37	Occipito-temporal cortex	0.220
				39	Angular Gyrus	0.206
(58, 18, 28)	2.89	0.0028	14	9	Dorsolateral prefrontal cortex	0.506
				45	pars triangularis, Broca’s area	0.238
				46	Dorsolateral prefrontal cortex	0.141
**Social** > **Non-social Joint Attention (Eye Contact effect)**						
(62, −50, 6)	3.81	0.00018	671	22	Superior Temporal Gyrus	0.479
				21	Middle Temporal Gyrus	0.382
				37	Occipito-temporal cortex	0.111
(−58, −70, 12)	3.76	0.00021	147	19	V3	0.335
				39	Angular Gyrus	0.314
				37	Occipito-temporal cortex	0.179
(−64, −48, 4)	2.76	0.00397	21	21	Middle Temporal Gyrus	0.512
				22	Superior Temporal Gyrus	0.429
(−60, −10, 40)	3.09	0.00159	32	6	Pre-motor and supplementary motor cortex	0.664
				3	Primary somatosensory cortex	0.135
(−48, −8, 40)	2.62	0.00574	16	6	Pre-motor and supplementary motor cortex	0.906
**Initiation of Joint Attention** > **Non-social Joint Attention (Eye Contact effect)**						
(48, −68, 16)	3.81	0.00018	443	39	Angular Gyrus	0.607
				19	V3	0.367
(42, −56, 58)	2.69	0.0048	17	7	Somatosensory Association Cortex	0.460
				40	Supramarginal Gyrus	0.441
(−32, −56, 56)	3.43	0.00059	284	7	Somatosensory Association Cortex	0.717
				40	Supramarginal Gyrus	0.242
(−56, −20, 42)	3.24	0.00103	112	6	Pre-motor and supplementary motor cortex	0.298
				2	Primary somatosensory cortex	0.243
				3	Primary somatosensory cortex	0.176
				1	Primary somatosensory cortex	0.165
(−58, 0, −4)	2.59	0.00615	13	21	Middle Temporal Gyrus	0.470
				22	Superior Temporal Gyrus	0.407
**Response to Joint Attention** > **Non-social Joint Attention (Eye Contact effect)**						
(64, −56, 16)	4.98	0	413	22	Superior Temporal Gyrus	0.380
				39	Angular Gyrus	0.241
				21	Middle Temporal Gyrus	0.170
				40	Supramarginal Gyrus	0.147
(58, −56, 32)	2.89	0.00279	27	40	Supramarginal Gyrus	0.605
				39	Angular Gyrus	0.351
(−58, −70, 10)	4.43	0.00002	394	19	V3	0.368
				39	Angular Gyrus	0.252
				37	Occipito-temporal cortex	0.196
				21	Middle Temporal Gyrus	0.103
(−58, −14, 12)	2.56	0.00668	21	22	Superior Temporal Gyrus	0.342
				42	Primary and Auditory Association Cortex	0.310
				43	Subcentral area	0.285
(−34, −70, 48)	2.62	0.00572	12	7	Somatosensory Association Cortex	0.735
				19	V3	0.203

*Clusters of positive activity for each contrast are listed. Horizontal lines separate results from each contrast (in bold). Negative x-coordinate indicates the left side. BA, Brodmann’s area. For each cluster in each contrast, the peak MNI coordinates, T-value, p-Value, anatomical labels, and probabilities are presented. MNI coordinates were converted to Tailarach coordinates to generate cluster labels.*

**TABLE 3:** Areas represented by the overlap (yellow area in Figure 7) of Figures 5, 6.

BA	Anatomical Area	Probability
39	Angular Gyrus	0.246
37	Occipito-temporal cortex	0.243
21	Middle Temporal gyrus	0.193
19	V3	0.177
22	Superior Temporal Gyrus	0.141

*Broadmann areas, anatomical labels and probabilities assigned using the Tailarach atlas.*

To further elucidate the effects of eye contact on initiating and responding to joint attention, results are presented using brain activity for the “initiation of joint attention > non-social joint attention” contrast, and for the “response to joint attention > non-social joint attention” contrast. For the “initiation > non-social” contrast, for each subject only the social runs in which that subject was the initiator were used, as well as the non-social runs. For the “response > non-social” contrast, for each subject only the social runs in which that subject was the responder were used, as well as the non-social runs. These analyses were possible because in each run, only one subject was the initiator for the entire duration of the run and the other subject was the responder. A contrast image was generated with these contrasts for each participant in the first-level analysis.

**FIGURE 8:**
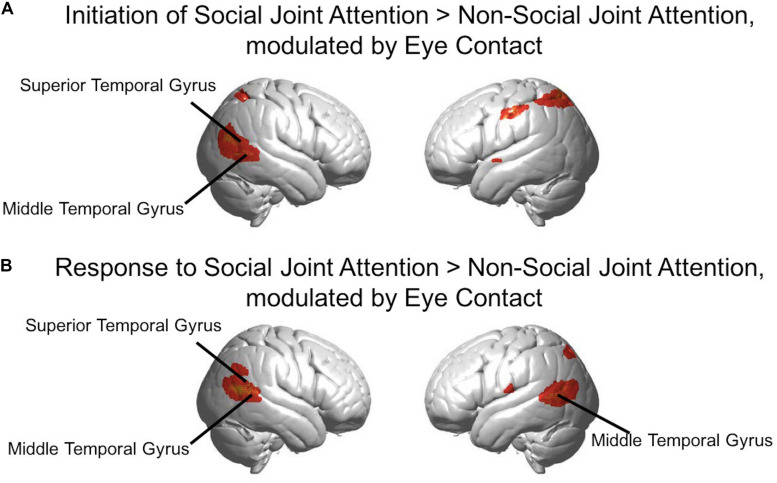
**(A)** Red-yellow areas on whole brain rendered images indicate greater activity in the initiation of joint attention > non-social joint attention, with greater activity in participants who made more eye contact (*p* < 0.01, uncorrected; *n* = 56). **(B)** Red-yellow areas on whole brain rendered images indicate greater activity in the response to joint attention > non-social joint attention for the social joint attention runs, with greater activity in participants who made more eye contact (*p* < 0.01, uncorrected; *n* = 56).

In the second level analysis, the modulatory effect of eye contact was calculated using the covariate regressor. Here, we present the result of the eye contact covariate regressor on the contrasts of Initiation > Non-social joint attention and Response > Non-social joint attention. The following clusters represent higher activity with greater eye contact for initiation of joint attention relative to the non-social condition (Figure 8A): the right angular gyrus [n of voxels = 443, t = 3.81, peak MNI coordinate of (48, −68, 16)], left somato-motor area [n of voxels = 112, t = 3.24, peak MNI coordinate of (−56, −20, 42)], and bilateral superior parietal areas [right: n of voxels = 17, peak t = 2.69, peak MNI coordinate of (42, −56, 58); left: n of voxels = 284, peak t = 3.43, peak MNI coordinate of (−32, −56, 56)]. The following clusters showed greater activity with higher amount of eye contact for the response to joint attention relative to the non-social condition (Figure 8B): right superior temporal gyrus [n of voxels = 413, peak t = 4.98, peak MNI coordinate of (64, −56, 16)], left angular gyrus [part of Wernicke’s area; n of voxels = 394, peak t = 4.43, peak MNI coordinate of (−58, −70, 10)], and bilateral parietal areas [right: n of voxels = 27, peak t = 2.89, peak MNI coordinate of (58, −56, 32); left: n of voxels = 12, peak t = 2.62, peak MNI coordinate of (−34, −70, 48)], along with smaller clusters in bilateral motor cortex. All whole-brain clusters are listed in Table 2. The results of the contrast of initiation of joint attention > response to joint attention with the group-level eye contact regressor are shown in the Supplementary Figure S3.

**FIGURE 9:**
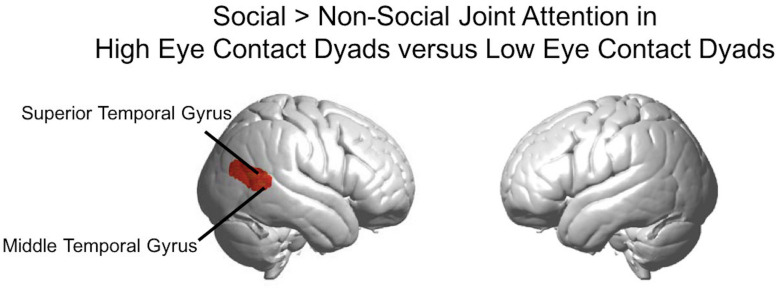
Social joint attention contrast in high eye contact dyads versus low eye contact dyads. Whole brain rendered images showing greater activity for social > non-social joint attention in the high eye contact dyads versus low eye contact dyads in right superior and middle temporal gyri (*p* < 0.01, uncorrected, *n* = 28).

The results above using the eye contact covariate regressor were calculated using the average number of eye contacts for each participant. We also split the participants into “high” and “low” eye contact dyads (blue and orange dots in Figure 3) to further illustrate the increase in the joint attention activity in participants who made more eye contact. The contrast of the social joint attention > non-social joint attention in the high eye contact dyads versus the low eye contact dyads resulted in an area of increased activity in the right superior and middle temporal gyrus (Figure 9), similar to the result of the eye contact frequency covariate on social > non-social joint attention for all dyads (Figure 6).

### Wavelet Coherence Results

**FIGURE 10:**
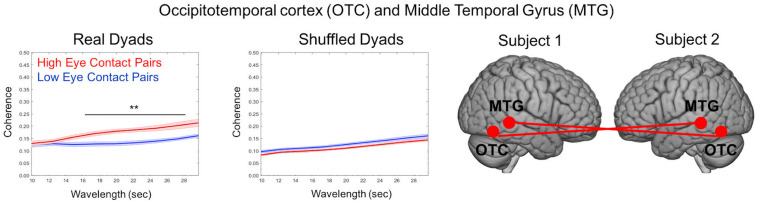
Cross-Brain Coherence Results. Coherence between occipito-temporal cortex (OTC) and middle temporal gyrus (MTG) of partners was increased in high eye contact pairs relative to low eye contact pairs (left graph). The coherence difference disappears when the pairs are shuffled (right graph). Graphs show coherence versus wavelength (averaged across time). Star indicates wavelengths at which difference was statistically significant (*p* < 0.05). Renderings indicate location of cohering brain regions, with each brain representing a participant in the dyad. Coherence was measured using bilateral areas (i.e., bilateral OTC to bilateral MTG).

One advantage of the dynamic two-person paradigm is the measure of neural coupling, which enables a test of the shared information hypothesis (Hasson and Frith, 2016). Cross-brain coherence was compared between high eye contact and low eye contact pairs for the social runs to test the specific hypothesis that increased frequency of eye-to-eye contacts will be associated with increased coherence between neural signals in regions that process face information. Coherence (Figure 10) is represented as the correlation between frequency components (*y*-axis) as a function of component wavelength (*x*-axis), with coherence between the high eye contact pairs in red and coherence between low eye contact pairs in blue. Coherence was increased (*p* < 0.05) between bilateral occipito-temporal cortex and middle temporal gyrus in the high eye contact group relative to the low eye contact group for wavelet components between 16 and 28 s. This was the only pair of areas that showed greater coherence for the high eye contact pairs than the low eye contact pairs. When the pairs were shuffled such that coherence was measured between people who did not do the task together, there was no significant difference between the coherence in the high eye contact versus low eye contact group.

## Discussion

Although the neural mechanisms of initiating and responding to joint attention have been previously studied using human neuroimaging techniques, few studies have examined the relationship between social joint attention and eye contact in a live, social context. Here we test the role of eye contact in joint attention. In the present study, simultaneous fNIRS hyperscanning and dual eye tracking were used to acquire neural signals in face-to-face interacting dyads, and to classify these dyads into groups based on the number of mutual eye contacts made prior to the direction of attention. Analyzing brain activity using frequency of eye contact as a covariate resulted in clusters of increased activity in the right superior temporal gyrus, middle temporal gyrus, and lateral occipito-temporal area, and in the left angular gyrus and lateral occipito-temporal cortex (Figure 6). In addition, coherence was greater across the middle temporal gyrus and occipito-temporal cortex in high eye contact dyads compared to low eye contact dyads.

Although the neural mechanisms of joint attention are well−studied, the relationship between joint attention with and without eye contact has not been investigated. Most studies of joint attention begin with eye contact between the initiator and responder, before the initiator shifts his/her gaze and the responder follows. However, in real world contexts, joint attention can be achieved with or without mutual eye contact as the initiating step. In fact, averted eye gaze is known to be a salient cue for directing the attention (Kleinke, 1986; Von Grünau and Anston, 1995; Senju et al., 2005; Conty et al., 2006). The main difference between joint attention that involves eye contact and joint attention that does not is whether the initiator’s eye gaze is first directed toward the responder or averted toward the target, respectively. The responder must always look at the initiator’s eyes in order to follow the gaze and achieve a joint attention event on the correct target. Additionally, some initiators made eye contact briefly with the responders again even after joint attention was achieved and before redirecting their attention to the object. Here, we show that accounting for this initiator behavior by analyzing the frequency of eye-to-eye contact is enough to replicate prior findings of increased activity in social and high-level visual networks. This is consistent with studies of direct versus averted eye gaze (Puce et al., 1998; Hoffman and Haxby, 2000; Pelphrey et al., 2004; Akiyama et al., 2006; Calder et al., 2007), further supporting the important role of the initiator in this behavior.

Direct gaze has also been shown in EEG studies to affect coherence between partners engage in interaction (Leong et al., 2017). In this study, joint gaze between infants and adults increased the neural coherence between them, is consistent with our coherence findings using functional NIRS. Here, we use wavelet coherence to relate the signals of interacting partners (Cui et al., 2012; Hirsch et al., 2017, 2018), which is done on the residual signals, after the task-related activity has been removed. Here, the task-related activity was due to the joint attention task, and this activity was removed prior to the cross-brain coherence analysis. We hypothesized that the residual fluctuations in the time series would be due behavioral events related to eye contact, and tested this by comparing the groups that made more eye contact with those who made less. Wavelet coherence assumes that sparse behavioral events result in similar short bursts of brain activity with a delay between interacting partners. Thus the finding of increased coherence between social and face-processing areas across high eye contact dyads compared to low eye contact dyads may indicate that some feature of eye contact modulates these small, sporadic neural events. For example, eye movements or eye blinks (Koike et al., 2019a) that occur during the eye contact events may be triggers of neural synchrony or social attention. The involvement of the middle temporal gyrus in the inter-brain coherence is consistent with the activity in this area in joint attention tasks (Redcay et al., 2012) as well as in eye contact experiments (Koike et al., 2016). The finding in the high eye contact group of increased coherence between the occipito-temporal cortex with the middle temporal gyrus is consistent with prior work showing that the occipito-temporal cortex is sensitive to eye contact and social interactions (Koike et al., 2016, 2019b; Piva et al., 2017). Koike et al. (2019a) have shown that occipito-temporal areas show greater inter-brain synchronization across partners making live eye contact compared to viewing a pre-recorded video (Koike et al., 2019b). A study in which participants played a game against a partner or against a computer showed greater cross-brain synchrony between the angular gyrus and the occipito-temporal area specific to the human-to-human interaction, where eye-to-eye contact contained relevant information (Piva et al., 2017). The findings of the current investigation contribute to this emerging literature consistent with increased neural coherence between social and high-level visual brain areas.

The right temporal parietal junction is an area that has been associated with many social functions, including theory of mind (Saxe and Kanwisher, 2003; Saxe and Wexler, 2005; Aichhorn et al., 2006; Perner et al., 2006), biological motion perception (Grossman et al., 2005; Pelphrey et al., 2005), and processing of dynamic faces and eyes (Puce et al., 1998; Langton et al., 2000; Pitcher et al., 2011). A functionally defined region, the TPJ subsumes aspects of the posterior superior temporal gyrus, angular gyrus, and supramarginal gyrus. Meta-analyses of functional imaging studies have shown that distinct parts of the TPJ may be responsible for different functions, and that eye gaze and social cognition in particular may be more restricted to the superior temporal gyrus (Carter and Huettel, 2013). The joint attention task described here involved direction of attention via eye gaze, and accounting for the initial mutual eye contact events in the analysis resulted in activity in different aspects of the right TPJ. Analysis of the task-related interaction activity resulted in a cluster that included the supramarginal gyrus. This is consistent with evidence showing that TPJ activity can be separated into anterior portion, including the supramarginal gyrus, that is more responsive to attention-direction, while the posterior portion is more sensitive to social cognition (Carter and Huettel, 2013). In the current study, activity in the supramarginal gyrus was not present when eye contact frequency was regressed in the group analysis; rather, the superior temporal and middle temporal gyri, and the lateral occipito-temporal cortex showed more eye contact-specific activity. These same areas also showed increased coherence across participants who made more eye contact, supporting the idea that eye contact was the driver of the cross-brain coherence.

The presented results are consistent with prior hyperscanning fMRI studies of interpersonal coherence in joint attention, eye contact, and other social tasks. Many of these studies have shown involvement and synchronization of both right superior temporal gyrus/sulcus and right inferior frontal gyrus. In one study, right posterior STS showed greater coherence across partners during both cooperation and competition, while right IFG showed more coherence with greater competition, modulated by empathy (Liu et al., 2017). Another study found increased cross-brain connectivity between the TPJ of partners during a cooperative task involving grip coordination (Abe et al., 2019). Studies using hyperscanning fMRI and joint attention tasks have also reported inter-brain coherence in the right TPJ (Bilek et al., 2015; Goelman et al., 2019), while another recent study demonstrated increased activity in the right anterior insular cortex (Koike et al., 2019a), an area from which signals can not be measured using fNIRS. Our findings of increased right STG/MTG activity in a cooperative joint attention task are consistent with this prior work. Other studies have demonstrated the importance of the right inferior frontal gyrus (IFG) alongside the right STG in studies of joint attention and eye contact (Saito et al., 2010; Tanabe et al., 2012; Caruana et al., 2015; Koike et al., 2016, 2019a). Here, we did not find right IFG activity, which may be due to the fewer number of uninstructed eye contact events that occurred during the task. It may be that if every pair had made eye contact during the joint attention task, this area would have shown more activity due to the eye contact itself or due to the inherent social traits of the participants. Further research is necessary to confirm how right IFG activity relates to eye contact in joint attention, especially in pairs who naturally make more eye contact.

These findings present a unique clinical significance in the study of disorders of social interaction, such as autism. It is well known that people with autism have difficulty both with initiating eye contact, as well as with joint attention. Results of neuroimaging on people with autism show decreased or altered neural activity when they engage in these behaviors (Senju and Johnson, 2009; Philip et al., 2012; Redcay et al., 2013). Our results indicate that if eye contact modulates the neural activity elicited by joint attention, the lack of spontaneous eye contact in this population may explain altered neural findings in joint attention.

Finally, we refer to the “social runs” as those in which there was an initiator and a responder of joint attention, and the responder had to follow the eye gaze of the initiator to direct their attention to the correct object. However, it could be argued that even during these runs, the low eye-contact dyads failed to engage in true social interaction or did not possess social traits present in the higher eye contact dyads, as measured by the frequency of eye contact. The responder in the low eye contact dyads essentially performed the task similarly during both run types, using the eye gaze as a cue in the social condition rather than the LED in the non-social condition. High eye contact dyads showed greater neural activity in social and visual areas during the social joint attention runs, in which they made eye contact, compared to the non-social joint runs, in which no eye contact was made. Performance did not differ in interaction or non-social runs but the interaction involved eye contact for this group. True social interaction could be defined as engagement of both individuals with each other, and it may be that the high eye contact dyads naturally interacted more as a result of individual trait differences. With this perspective, it is not surprising that the high eye contact dyads showed more recruitment of hemodynamic activity to social and high-level visual brain regions, as well as more coherence between these same networks across interacting partners, as these dyads engaged in true social interaction.

In this study, amount of eye contact was used as a measure of social behavior that varied between dyads. We assume that this behavior is largely driven by differences in individual traits, that are amplified by the social context. For example, a naturally shy person would most likely not make as much uninstructed eye contact when initiating joint attention trials with his/her partner. We did not collect behavioral measures of socialness and comfort with social interactions, such as self-reported standardized social anxiety scales, from the participants. It is possible that the individual trait differences drove the number of eye contacts made during the interaction and the subsequent increases in neural activity in social and high-level visual brain areas. In addition to individual traits, other aspects of the interaction may also have influenced the brain activity of the participants. For example, some pairs of participants may have found each other mutually attractive, or felt more engaged with each other for some reason than other pairs. Further work including behavioral measures of “socialness” within individuals and “interactivity” between participants is needed to elucidate the specific traits and behaviors of “more social” people and “more connected” partners, and how these traits and behaviors affects neural activity during joint attention with a partner.

Using optical imaging methods and computational techniques for dual-brain and multi-modal imaging, we show that eye contact modulates the social system that engages in the neural activity related to joint attention, and that interacting partners who engage in more eye contact show more coherence between social and eye-gaze processing brain regions. These results suggest that some aspect of eye contact may contribute to the interaction involved in directing another person’s gaze toward an object. Further studies using hyperscanning as well as simultaneous acquisition of behavioral measures are necessary to confirm whether eye movements or some other aspect of face or eye-processing drives this brain activity, and how it relates to the observation of coherence across individuals that is specific to pairs who make more eye contact.

### Limitations

A common finding in neuroimaging studies of joint attention is activity in the medial prefrontal cortex (Schilbach et al., 2010; Redcay et al., 2012, 2013) and in reward-related regions in the basal ganglia (Schilbach et al., 2010) when participants engage in joint attention. Hemodynamic activity acquired by fNIRS can only be detected from the superficial cortex. It is likely that we did not detect joint attention-related activity in the mPFC and basal ganglia due to our inability to record from these areas.

While the results reported here are consistent with a large body of prior work exploring the neural mechanisms of joint attention, it should be noted that no multiple comparisons correction was applied to either the intra-brain GLM or the inter-brain coherence analysis. By using a combination of the oxyhemoglobin and deoxyhemoglobin signals and restricting the number of wavelengths in the coherence analysis, we reduce the likelihood of false positive results. However, this possibility cannot be ruled out with the thresholds reported here; further work is necessary to confirm and validate these descriptive findings of the modulatory effect of live eye contact on joint attention.

The small area of overlap (Figure 7) between the brain regions represented in Figures 5, 6 may represent an area of the brain sensitive to both the mechanisms of social joint attention as well as the modulation of eye contact on these mechanisms. A future study using separate eye contact and joint attention tasks could confirm whether this region is significantly activated by both tasks.

Finally, we report the modulatory effect of eye contact on social joint attention using the average number of mutual eye contact events across all social runs for each participant. An important question arises from this work is what is the direct effect of a mutual eye contact event that precedes a joint attention event? While the answer to this question is beyond the scope of this manuscript, it is an important future direction of this work.

## Data Availability Statement

The datasets generated for this study are available on request to the corresponding author.

## Ethics Statement

The studies involving human participants were reviewed and approved by the Yale University Human Investigation Committee (HIC # 1501015178). The patients/participants provided their written informed consent to participate in this study. Written informed consent was obtained from the individual(s) for the publication of any potentially identifiable images or data included in this article.

## Author Contributions

SD designed and performed the experiment, and analyzed the data. SD and JH wrote the manuscript. JN and XZ assisted with planning, data acquisition, and analysis.

## Conflict of Interest

The authors declare that the research was conducted in the absence of any commercial or financial relationships that could be construed as a potential conflict of interest.
