# Supplementary material for: Joint Attention During Live Person-to-Person Contact Activates rTPJ, Including a Sub-Component Associated With Spontaneous Eye-to-Eye Contact
Source: Front Hum Neurosci. 2020 Jun 3;14:201. doi: 10.3389/fnhum.2020.00201 (PMC7283505; doi:10.3389/fnhum.2020.00201)
Supplement: Supplementary file 1 [file Table_1.docx]

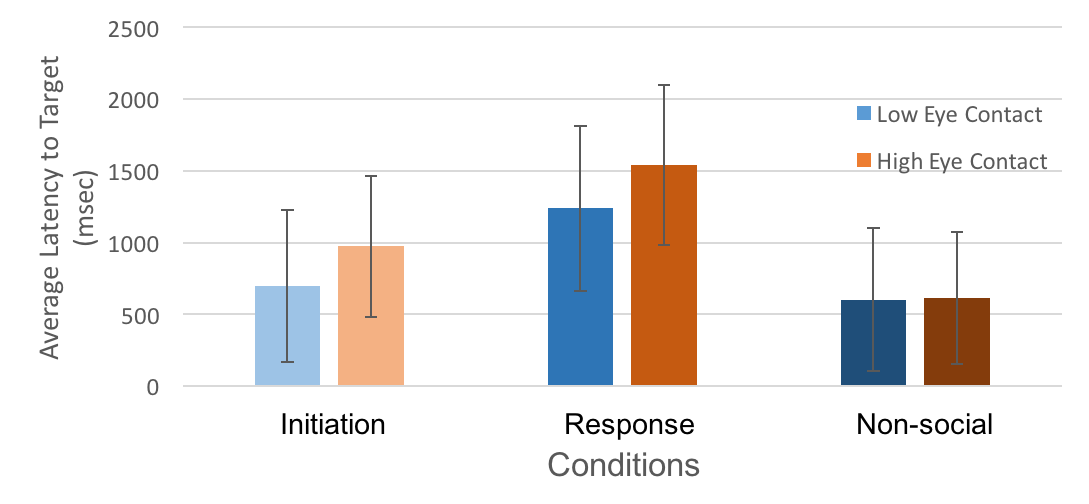


**Figure S1.** Average latency to target by condition and group. Asterisks indicate significant paired t-test (one star: p<0.05; two: p<0.01; three: p<0.001, four: p<0.0001). Participants took longer to direct eye gaze to target when they were the responder in the social runs than when they were the initiator. Participants were fastest in directing attention to target during the non-social runs when they received an LED cue than when they were the responder during the social runs. The high eye contact group also took longer to look at the target than the low eye contact group (F(1, 147)=6.86; p=0.0092). There was no significant group by condition interaction.


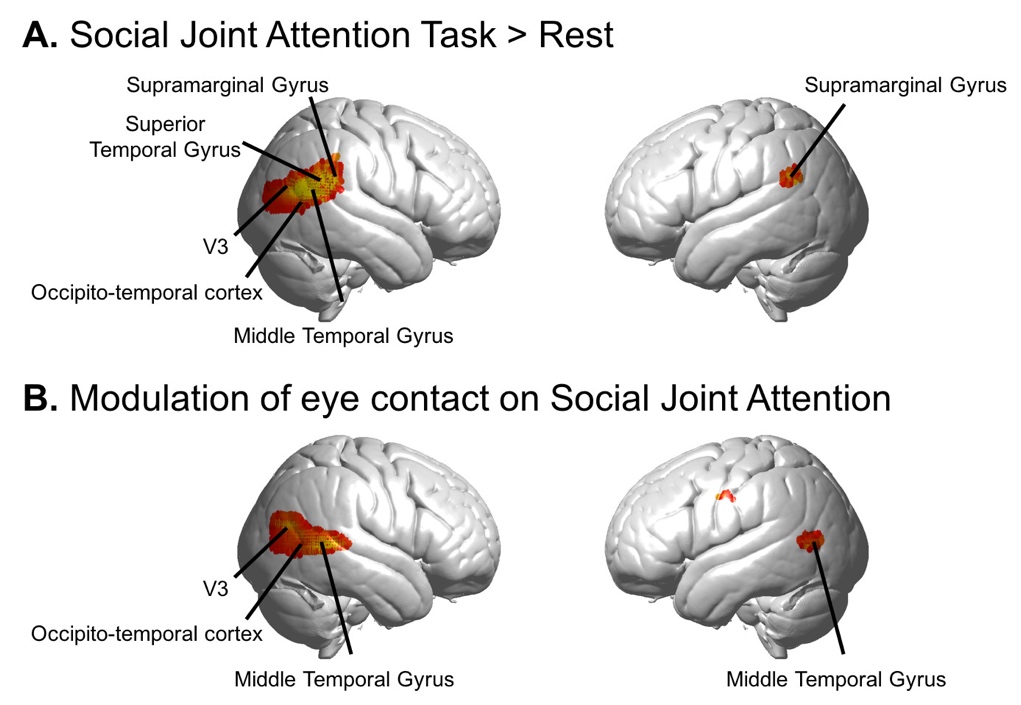


**Figure S2**. Social joint attention task relative to rest. Whole brain rendered images showing greater activity for social joint attention task > rest (p<0.01, uncorrected). Social conditions include all runs in which one subject was an initiator and one was a responder. Top row shows task > rest main effect. Bottom row shows the modulation of eye contact on the social joint attention task. Results are combined across all participants (n=56).


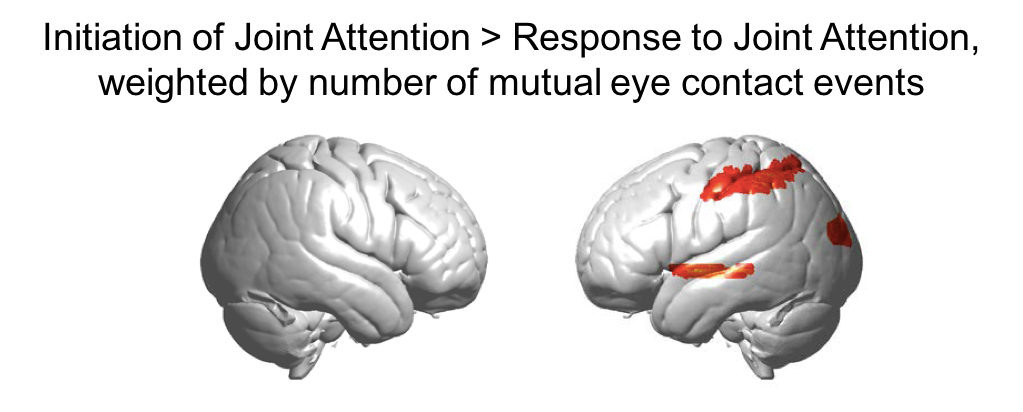


**Figure S3**. GLM Contrast of Initiation of joint attention > Response to joint attention, with the covariance by the average number of mutual eye contact events for each participant in the group-level analysis (p<0.01, uncorrected, n=56).
